# Murid Herpesvirus-4 Exploits Dendritic Cells to Infect B Cells

**DOI:** 10.1371/journal.ppat.1002346

**Published:** 2011-11-10

**Authors:** Miguel Gaspar, Janet S. May, Soumi Sukla, Bruno Frederico, Michael B. Gill, Christopher M. Smith, Gabrielle T. Belz, Philip G. Stevenson

**Affiliations:** 1 Division of Virology, Department of Pathology, University of Cambridge, Cambridge, United Kingdom; 2 Division of Immunology, The Walter and Eliza Hall Institute of Medical Research, Melbourne, Victoria, Australia; Emory University, United States of America

## Abstract

Dendritic cells (DCs) play a central role in initiating immune responses. Some persistent viruses infect DCs and can disrupt their functions *in vitro*. However, these viruses remain strongly immunogenic *in vivo*. Thus what role DC infection plays in the pathogenesis of persistent infections is unclear. Here we show that a persistent, B cell-tropic gamma-herpesvirus, Murid Herpesvirus-4 (MuHV-4), infects DCs early after host entry, before it establishes a substantial infection of B cells. DC-specific virus marking by cre-lox recombination revealed that a significant fraction of the virus latent in B cells had passed through a DC, and a virus attenuated for replication in DCs was impaired in B cell colonization. *In vitro* MuHV-4 dramatically altered the DC cytoskeleton, suggesting that it manipulates DC migration and shape in order to spread. MuHV-4 therefore uses DCs to colonize B cells.

## Author Summary

We detect invading viruses with dendritic cells and eliminate them with lymphocytes. A key interaction is lymphocyte activation by dendritic cells presenting viral antigens. Not all viruses can be eliminated, and some that persist deliberately colonize lymphocytes and dendritic cells, such that parasitism and host defence co-exist within the same sites. Once established, these infections are very hard to eliminate. Therefore to vaccinate against them we must determine how infection first occurs. Here we show that a gamma-herpesvirus relation of the Kaposi's Sarcoma-associated Herpesvirus and Epstein-Barr virus - B cell-tropic human pathogens that cause cancers - uses dendritic cells to reach and infect B lymphocytes. Dendritic cells were infected before B cells; viruses marked genetically in dendritic cells were recovered from B cells; and a virus unable to replicate in dendritic cells infected B cells poorly. Thus dendritic cells not only present viral antigens to lymphocytes, but can be exploited by evasive viruses to infect lymphocytes. Therefore targeting dendritic cell infection could be an effective means of vaccine-primed host defence.

## Introduction

Dendritic cells (DCs) act as sentinels against infection: they encode pathogen-responsive receptors, abound at pathogen entry sites, and orchestrate both innate and adaptiveimmune responses [1], [2]. Virus-infected DCs are generally immunogenic [3]–[5], and DC infection may be important for optimal T cell priming [6]. However several persistent viruses, which might be expected to limit their exposure to host immunity, efficiently infect DCs [7]–[10]. The infected DCs may function abnormally *in vitro* [11], [12], but the corresponding *in vivo* infections remain potently immunogenic. Therefore how DC infection benefits these persistent viruses, or whether it instead benefits the host, is unclear.

Murid Herpesvirus-4 (MuHV-4) is a gamma-herpesvirus that readily allows *in vivo* analysis of host colonization [13], [14]. Like Epstein-Barr virus and the Kaposi's Sarcoma-associated Herpesvirus, MuHV-4 persists in B cells [15], [16]. It also acutely infects macrophages and dendritic cells [17]–[19]. Myeloid infection provides MuHV-4 with a site of persistence in B cell-deficient mice, but the antibody deficiency of these mice leads to a somewhat atypical chronic lytic infection [20]; in immunocompetent mice, infected macrophages and dendritic cells are hard to detect long-term [19]. Therefore myeloid infection seems more likely to be important for establishing MuHV-4 host colonization than for maintaining it. Epstein-Barr virus and Kaposi's Sarcoma-associated Herpesvirus can also infect myeloid cells *in vitro* [9], [21]. While they seem rarely to do so *in vivo*, their clinical presentations post-date infection by at least 1 month [22], so early host colonization is rarely studied. Thus acute myeloid cell infection may not be unique to MuHV-4.

The difficulty of curing established gamma-herpesvirus infections makes early events in host colonization important to understand. It has been suggested that incoming Epstein-Barr virus infects B cells and so establishes latency directly [23]. This would argue against an important role for myeloid infection. However, good supporting evidence for direct B cell infection is lacking. Indeed vaccination to block Epstein-Barr virions binding to B cells failed to reduce the incidence of infection [24]. And while DNA from lung-inoculated, replication-deficient MuHV-4 has been found associated with B cells [25], [26], viral genome-positive B cells did not reach the spleen, so whether the detected DNA was a viable primary infection or merely adsorbed debris was unclear. That fibroblast-propagated MuHV-4 infects mice well [27] and B cells poorly [28] would argue against B cells being a significant primary target. Natural host entry probably occurs via the upper respiratory tract rather than the lung [29], but MuHV-4 lacking thymidine kinase or ribonucleotide reductase fails to infect by this route and neither enzyme is required for replication in B cells [30], [31]. Therefore here too B cells would seem an unlikely primary target.

How then does MuHV-4 reach B cells? HIV can infect T cells via DCs [32], [33], and DCs also communicate with B cells [34], so exploiting DC/lymphocyte interactions could be a common theme among lymphotropic viruses. However, *in vivo* evidence is again sparse. HIV-infected DCs are hard to find *in vivo*, and most HIV taken up by DCs *in vitro* is degraded. Thus it has been possible only to hypothesize that DCs contribute to host entry [35]. MuHV-4 allows more comprehensive analysis, and this is what we undertook here. As removing DCs causes immunosuppression [36], we used DC-specific cre recombinase expression to inhibit MuHV-4 replication in DCs or to mark genetically viruses that had replicated in a DC, while leaving normal DC functions intact.

## Results

### Direct visualization of MuHV-4 spread

**Figure 1:**
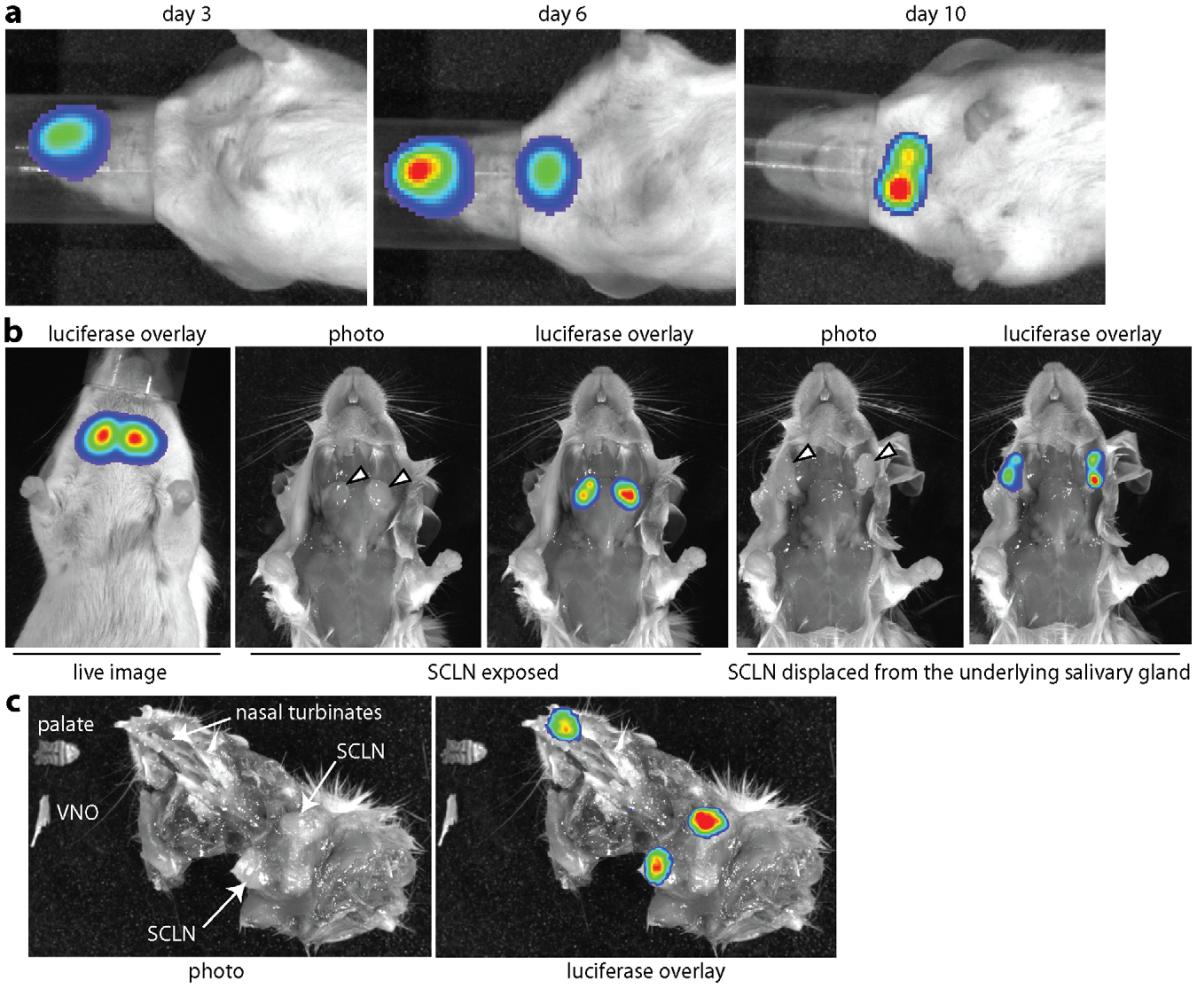
Direct visualization of lymph node colonization after non-invasive MuHV-4 infection. a. Mice were infected with MuHV-4 expressing luciferase from a viral early lytic promoter (10^4^ p.f.u. intranasally without anaesthesia). Infection was then tracked by injection with luciferin and CCD camera scanning. Equivalent data were obtained with more than 20 mice. **b.** The live imaging signal at day 10 post-infection was localized to the superficial cervical lymph nodes (SCLN, indicated by arrowheads). No signal was observed from the underlying salivary glands. **c.** Dissection at day 6 post-infection revealed luciferase signals in the nose and SCLN but not intervening sites. A ventral view of the head and neck is shown, with the lower jaw, vomeronasal organ (VNO) which lies ventral to the nasal turbinates, and palate which lies posterior to them, removed. The palate and VNO are shown separately.

That MuHV-4 colonizes B cells is indisputable, but where it first infects them is unclear. To identify the likely anatomical site we used viral luciferase expression [29] to track host colonization after upper respiratory tract infection (Fig. 1). Live imaging signals were evident in noses 3 days after inoculation and in the neck after 10 days (Fig. 1a). Signals were present in both sites after 6 days, indicating that the virus spread from nose to neck at 3–6 days post-infection.

*Ex vivo* imaging of dissected tissues at the peak of neck infection - day 10 - (Fig. 1b) established that this signal came entirely from the superficial cervical lymph nodes (SCLN), which receive lymphatic drainage from the nose [37]. No other tissues were luciferase^+^ at this time. Imaging dissected mice at day 6 (Fig. 1c) revealed luciferase expression in the nasal turbinates and in the SCLN, but not in intervening sites. Nor was luciferase expression evident in other lymph nodes, or in the nasal-associated lymphoid tissue that lies on either side of the palate. Therefore MuHV-4 appeared to move directly from the nose to the SCLN.

### Host colonization measured by viral DNA loads and recoverable infectivity

**Figure 2:**
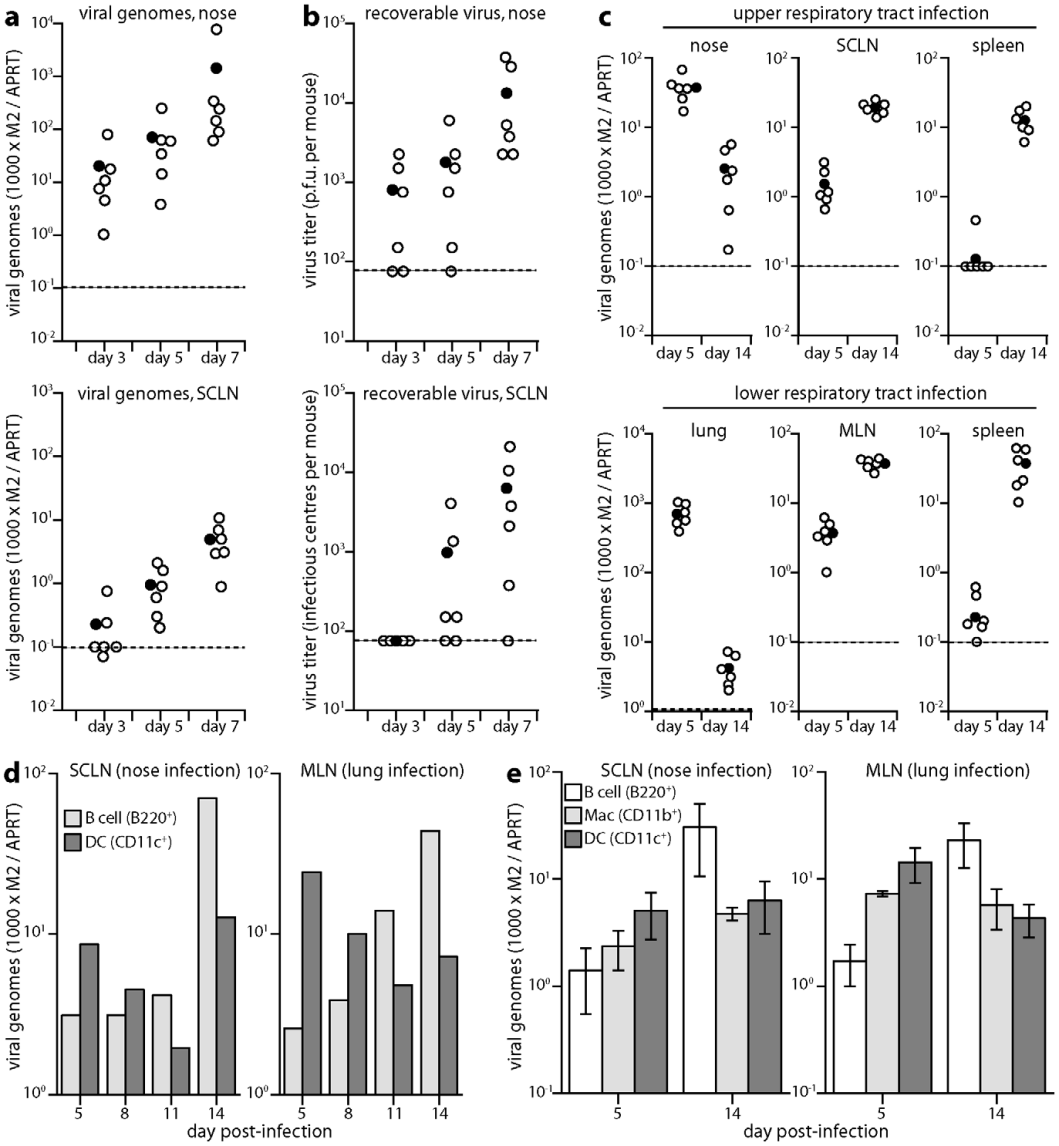
Host colonization tracked by Q-PCR. a. DNA was extracted from noses and SCLN at different times after upper respiratory tract infection (10^4^ p.f.u.), and viral copy number determined by Q-PCR. Viral copy numbers (M2) were normalized according to the cellular copy number (APRT) of the same sample. Open circles show individual mice (n = 6); filled circles show means; dashed lines show lower limits of assay sensitivity. Equivalent data were obtained in 3 replicate experiments. **b.** The same nose and SCLN samples were analyzed for recoverable virus by plaque assay (noses) and infectious centre assay (SCLN). Open circles show individual mice; filled circles show means; dashed lines show assay sensitivity limits. Equivalent data were obtained in 2 replicate experiments. **c.** During either early lytic replication (day 5) or virus-driven lymphoproliferation (day 14) after nose or lung infections (10^4^ p.f.u.), viral loads were determined by Q-PCR of the peripheral replication site (nose or lung), draining lymph nodes (SCLN or MLN) and spleen. At day 5, SCLN and mediastinal lymph node (MLN) viral loads were significantly higher than those in spleens (p<0.001 by Student's 2-tailed t test). At day 14 they were equivalent. Equivalent data were obtained in 2 replicate experiments. **d.** Mice were infected (10^4^ p.f.u.) in either noses or lungs. SCLN and MLN were then pooled from 3 mice per time point and separated into B cells and DCs on affinity columns. Each population was analyzed for viral DNA by Q-PCR. Viral copy numbers (M2) are expressed relative to the cellular DNA copy number (APRT). Equivalent data were obtained in 2 replicate experiments. **e.** Mice were infected as in **d**. Lymph nodes were pooled from 3 mice per group at each time point and separated on affinity columns into B cells, DCs and macrophages. Each bar shows mean ± SEM of 3 experiments. For both nose and lung infections, B cell genome loads were significantly high than those of DCs at day 14 and significantly lower at day 5 (p<0.05 by Student's t test). Viral genome loads were also higher in macrophages than in B cells at day 5 after lung infection (p<0.05), but not at day 5 after nose infection (p = 0.4).

We next tracked infection by quantitative PCR (Q-PCR) of viral DNA (Fig. 2a). In agreement with the luciferase imaging, viral genomes were detected in the nose at day 3 post-inoculation and not consistently in the SCLN until day 5. Recovering replication-competent virus by infectious centre assay (Fig. 2b) was less sensitive but showed similar trends, arguing that the DNA signals in the SCLN were due to infection rather than just accumulated viral debris. Both upper and lower respiratory tract infections showed viral genomes being most abundant in the peripheral epithelial site at day 5 post-inoculation, and most abundant in lymphoid tissue at day 14 (Fig. 2c). At day 5 viral genomes were more abundant in draining lymph nodes than in spleens, whereas by day 14 these sites were equivalent. These results were consistent with epithelial infection seeding first to its draining lymph nodes, and then disseminating to the spleen, presumably via B cells [38].

### Kinetic comparison of DC and B cell infections

Previous analyses of MuHV-4 genome loads in different cell types have focussed on peak splenic titers, when most infected cells are B cells [18], [19]. We reasoned that if B cells only became infected once virus reached lymph nodes, then early on viral genome loads might be lower in lymph node B cells than in the cells first responsible for taking the virus there. Antigen transport of lymph nodes is a major function of DCs. We therefore tested whether DC infection might precede B cell infection, by separating these sub-populations from acutely infected lymph nodes on affinity columns and determining their viral genome loads by Q-PCR (Fig. 2d). At days 11 and 14 after virus inoculation into either noses or lungs, viral genome loads were higher in lymph node B220^+^ cells (B cells) than in CD11c^+^ cells (DCs), but at days 5 and 8 they were higher in DCs.

Further analysis (Fig. 2e) identified significantly higher viral genome loads also in CD11c^-^CD11b^+^ lymph node cells (macrophages) than in B cells early after virus inoculation into the lungs, but not after virus inoculation into the nose. At 8 days after virus inoculation into the upper respiratory tract, 10^5^ cells purified from the SCLN of pooled pairs of mice yielded 20.0±7.6 infectious centres for macrophages, 53.3±26.2 for DCs and 14.7±4.3 for B cells (mean ± SEM, n = 3). Each population yielded <1 p.f.u. per 10^5^ cells by plaque assay. Therefore after upper respiratory tract infection, a predominantly latent infection of DCs appeared to precede that of B cells. SCLN suspensions are typically 50% B cells and <5% DCs, so at all time points B cells accounted for most of the recoverable viral DNA. However virus seeds to the SCLN at a low level, is rapidly passed to B cells, which proliferate, and seeds asynchronously between individual mice. Thus by the time DC infection is readily detected, some B cell infection and amplification has inevitably occurred. The kinetic changes in relative genome load argued strongly for DC infection preceding B cell infection: the genome^+^ DCs at day 14 may have acquired infection from B cells, but this was unlikely at day 5 because B cell viral loads then were low.

### Identification of MuHV-4-infected DCs

**Figure 3:**
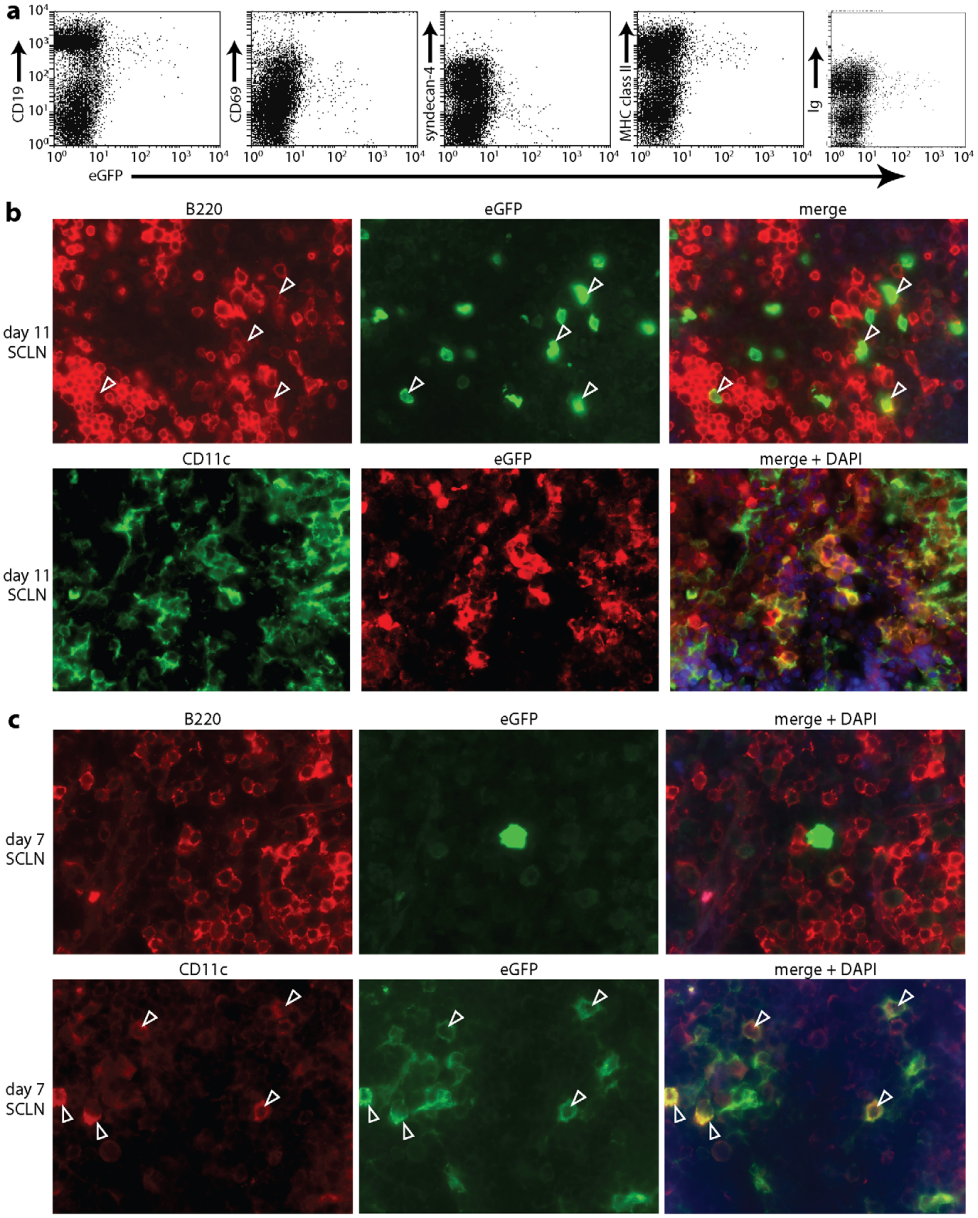
Direct visualization of MuHV-4-infected DCs in lymph nodes. a. Mice were infected in the nose (10^4^ p.f.u.) with MuHV-4 expressing eGFP from an intergenic EF1α promoter. Flow cytometric analysis of SCLN cells 11 days later established that >90% of eGFP^+^ cells were CD19^+^, immunoglobulin^+^ and MHC class II^hi^. >70% were CD69^+^ and >80% were syndecan-4^lo^. Equivalent data were obtained in 3 replicate experiments. **b.** After an equivalent infection, SCLN sections were co-stained for eGFP and either the B cell marker B220 or the DC marker CD11c. Both eGFP^+^B220^+^ (arrowheads) and eGFP^+^CD11c^+^ cells were readily identifiable. **c.** SCLN sections analyzed as in **b** but at 7 days post-infection showed that the eGFP^+^ cells were almost all CD11c^+^ and not B220^+^. The arrowheads show examples. Equivalent data were obtained in 2 replicate experiments. Note that the sections show representative staining patterns rather than the total level of staining, which was always greater at day 11 than at day 7.

We sought next to visualize infected cells directly, using MuHV-4 that expresses eGFP from an intergenic EF1α promoter [39] (Fig. 3). At day 11 post-infection - that is after the onset of virus-driven lymphoproliferation - flow cytometry identified viral eGFP expression mainly in B cells (Fig. 3a): approximately 1% of CD19^+^ lymph nodes cells were eGFP^+^. The eGFP^+^ cells were also positive for surface immunoglobulin and MHC class II. Most were CD69^+^, consistent with MuHV-4 up-regulating this acute activation marker on B cells [40]. B cells are normally syndecan-4^+^ [41], so a surprising finding was that most eGFP^+^ cells were syndecan-4^-^. Syndecan-4 is a carrier of heparan sulfate, on which MuHV-4 infection strongly depends [42], so MuHV-4 may down-regulate syndecan-4 on infected B cells to prevent super-infection.

No clear CD19^-^eGFP^+^ population was identifiable by flow cytometry. However, immunohistochemistry at day 11 post-infection identified both eGFP^+^B220^+^ and eGFP^+^CD11c^+^ cells in lymph nodes (Fig. 3b). At day 7 (Fig. 3c) eGFP^+^ cells in the SCLN were 64.0±13.0% CD11c^+^, 14.1±5.1% B220^+^ and 21.9±12.3% neither (mean ± SEM, n = 6 mice, counting >100 eGFP^+^ cells per mouse). At day 11, SCLN eGFP^+^ cells were 12.0±2.8% CD11c^+^, 78.0±2.4% B220^+^, and 10.0±3.1% neither. Therefore both PCR of viral DNA and immunostaining of virus-expressed eGFP showed DC infection early in lymph node colonization, before B cell infection was well established. Our failure to detect a clear population of infected DCs by flow cytometry possibly reflected that these cells are difficult to isolate intact.

### A marker virus for assaying exposure to cre recombinase

**Figure 4:**
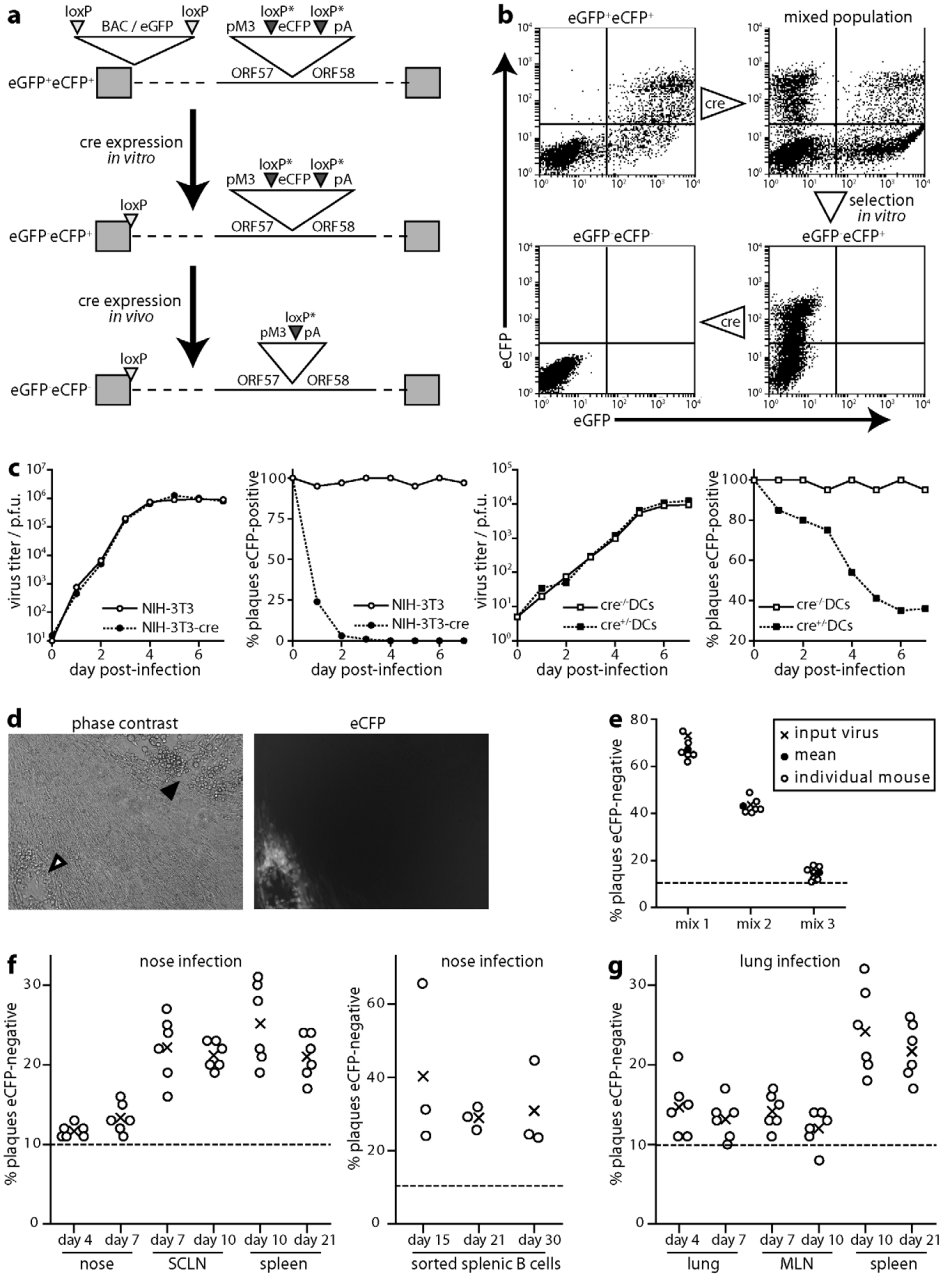
Cre/lox marking of MuHV-4 replicating in DCs. a. The BAC/eGFP cassette at the left end of the MuHV-4 genome is flanked by loxP sites. An additional eCFP expression cassette with variant loxP sites was inserted between ORFs 57 and 58. After exposure to limited cre recombinase expression, eCFP^+^eGFP^-^ viruses could be selected and then assayed for further cre exposure by eCFP loss. **b.** As outlined in **a**, NIH-3T3-cre cells were infected (18 h, 2 p.f.u./cell) with eGFP^+^eCFP^+^ MuHV-4, and eCFP^+^eGFP^-^ viruses selected from the resulting mixed population by flow cytometric sorting of infected cells and limiting dilution cloning of the virus produced. Further virus exposure to cre by low multiplicity NIH-3T3-cre cell infection led to eCFP loss. Note that eGFP is driven by an Rta-independent HCMV IE1 promoter and eCFP by an Rta-dependent M3 promoter, so latent or immediate early lytic infection can be eGFP^+^eCFP^-^; with plaque typing this was not an issue because most infection is late lytic. **c.** ECFP^+^eGFP^-^ MuHV-4 was tested for growth and loss of eCFP expression in NIH-3T3 fibroblasts and bone marrow-derived DCs, each with or without cre expression. Infected cell supernatants were titered by plaque assay and plaques scored as eCFP^+^ or eCFP^-^ under ultra-violet illumination. Equivalent data were obtained in 2 replicate experiments. **d.** Examples of eCFP^+^ and eCFP^-^ plaques recovered by infectious centre assay from cre^+^ mice infected with eCFP^+^eGFP^-^ MuHV-4. **e.** Cre^-^ mice were infected in the nose with different mixtures of eCFP^+^ and eCFP^-^ MuHV-4 (total 10^4^ p.f.u.). Viruses were recovered 20 days later by infectious centre of spleen cells and typed for eCFP expression in parallel with a sample of the original inoculum. Equivalent data were obtained in 2 replicate experiments. **f.** CD11c-cre^+^ mice were infected in the upper respiratory tract with eCFP^+^eGFP^-^ MuHV-4 (10^4^ p.f.u.). Viruses were then recovered from noses by plaque assay, and from lymph nodes and spleen by infectious centre assay, and scored as eCFP^+^ or eCFP^-^. Circles show individual mice; crosses show means. The proportions of eCFP^-^ plaques were significantly above background (<5% eCFP^-^) for both noses and lungs (p<0.01 by Student's t test), significantly above noses for SCLN (p<0.03) and significantly above noses or lungs for spleens (p<0.01), but not significantly above lungs for MLN (p = 0.4). Equivalent data were obtained in 2 replicate experiments. **g.** CD11c-cre^+^ mice were infected as in **f**, but in the lower respiratory tract. Viruses were recovered from lungs by plaque assay, and from lymph nodes and spleen by infectious centre assay.

Cre-lox recombination allows transient infection events to be recorded by a permanent genetic mark [43]. We used this technology to identify MuHV-4 that had replicated in cre recombinase^+^ cells, by inserting a loxP-flanked eCFP expression cassette between the 3′ ends of ORFs 57 and 58 (Fig. 4a). The MuHV-4 BAC/eGFP cassette is also flanked by loxP sites [44], so BAC-derived viruses retain a single loxP site at the genome left end. To avoid recombination between this site and those flanking eCFP, we incorporated a point mutation into the spacer region of the latter. We then transfected BAC DNA into BHK-21 cells, passed the recovered eGFP^+^eCFP^+^ virus once through cre^+^ NIH-3T3 cells (2 p.f.u./cell) and selected eGFP^-^eCFP^+^ virus clones from the mixed progeny (Fig. 4b).

LoxP-eCFP MuHV-4 (eGFP^-^eCFP^+^) showed no *in vivo* growth deficit (Fig. S1). *In vitro* it rapidly lost eCFP expression when passed through cre^+^ NIH-3T3 cells (Fig. 4c). We quantitated this loss by counting plaques under phase contrast and typing each plaque as eCFP^+^ or eCFP^-^ under ultra-violet illumination (Fig. 4d). LoxP-eCFP MuHV-4 also lost eCFP expression in cre^+^DCs - derived from CD11c-cre transgenic mouse bone marrow by growth in GM-CSF - albeit less dramatically than in cre^+^ fibroblasts (Fig. 4c). Thus eCFP loss provided a minimum estimate of the proportion of virions passing through a DC.

### Functional evidence for MuHV-4 reaching B cells via DCs

We looked for a possible selective effect of eCFP excision by infecting non-transgenic (cre^-^) mice with different mixtures of eCFP^+^ and eCFP^-^ derivatives of the loxP-eCFP virus, and 20 days later typing splenic infectious centres for eCFP expression (Fig. 4e). No marked difference in eCFP^+^ frequency was observed between the input and recovered viruses. ECFP loss therefore provided an unbiased marker of *in vivo* virus exposure to cre. A limitation of eCFP-based analysis was that even non-transgenic mice yielded 6.1±2.8% eCFP^-^ plaques (mean±SD, n = 12). PCR across the ORF57/58 junction identified loxP recombination in 6/6 eCFP^-^ plaques recovered from CD11c-cre mice infected with loxP-eCFP MuHV-4. 3/3 eCFP^-^ plaques recovered from non-transgenic mice (which were rare) did not show loxP recombination, and were presumably due to cassette silencing: that viral expression cassettes are not always active even in lytically infected cells is well-established [45]. Therefore we took 10% eCFP^-^ plaques (>1 SD above the background) as indicating significant virus exposure to cre recombinase.

We then infected CD11c-cre transgenic mice with loxP-eCFP MuHV-4 in the upper respiratory tract. The infectious virus recovered from noses showed little eCFP loss above the 10% cut-off (Fig. 4f). However eCFP loss from SCLN virus was significantly greater. Therefore DCs contributed relatively little to peripheral viral lytic replication, and much more to SCLN colonization. Splenic virus showed no further eCFP loss, consistent with the spread from the SCLN to here being accomplished by B cells. Substantial eCFP expression loss from the viruses of flow cytometrically sorted B cells (Fig. 4f) established that a significant proportion of the virus establishing long-term latency had passed through a DC.

The relative inefficiency of cre-mediated eCFP excision in CD11c-cre mice made it difficult to estimate exactly what proportion of the virus in B cells had passed through a DC. To answer this better we exposed DCs grown from CD11c-cre bone marrow *in vitro* to MuHV-4 (5 p.f.u./cell, 6 h, 37°C), then washed them in pH = 3 buffer to inactivate non-endocytosed virions and 24 h later - the earliest new infectivity can be recovered from MuHV-4-exposed DCs - titrated cell supernatants by plaque assay. ECFP typing under ultra-violet illumination identified 14.2±3.0% of plaques as eCFP^-^ (mean ± SD of 10 cultures, counting at least 100 plaques for each culture). Thus assuming that an overnight infection of bone marrow-derived DCs equates roughly in cre exposure to DC-mediated *in vivo* virus transfer, essentially all the virus derived from B cells after upper respiratory tract infection had passed through a DC.

The virus recovered from lungs after lower respiratory tract infection (Fig. 4g) showed only modest eCFP loss. Unlike after upper respiratory tract infection, there was no increased eCFP loss upon reaching the draining lymph nodes (MLN). Therefore lung infection appeared to provide MuHV-4 with an alternative, DC-independent route to lymphoid tissue. Viruses recovered from spleens after lung infection showed more eCFP loss. However, nose infections inevitably accompany lung infections, so the splenic virus would also have come from the SCLN. Thus while DCs took virus to lymph nodes from the upper respiratory tract, they were less important when a more invasive entry point was used.

### Cre-mediated virus inactivation

**Figure 5:**
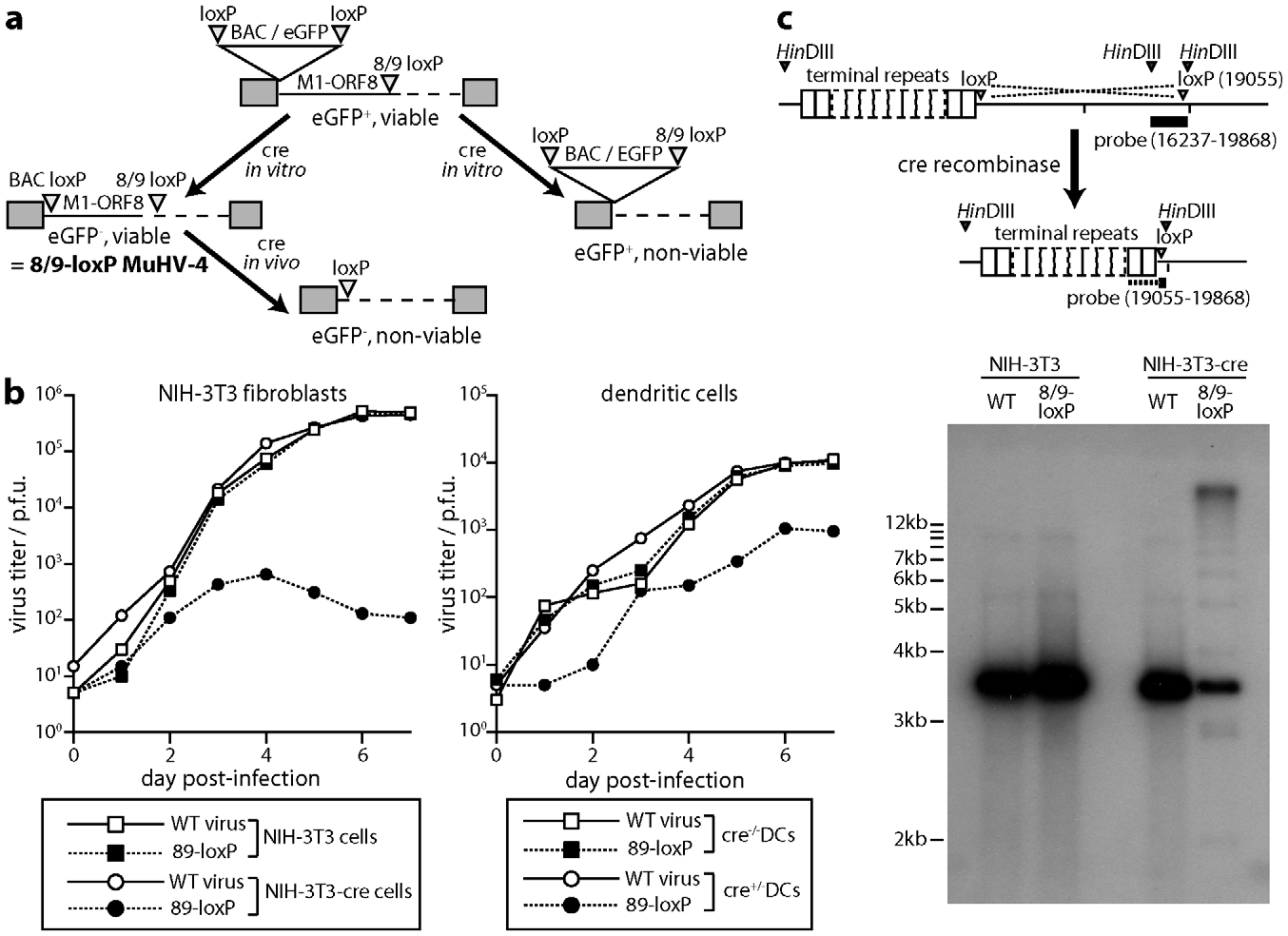
Virus inactivation by cre/lox recombination. a. A loxP site compatible with those flanking the BAC/eGFP cassette was inserted between MuHV-4 ORFs 8 and 9. After a single passage through NIH-3T3-cre cells (2 p.f.u./cell), we selected viable eGFP^-^ virus clones (8/9-loxP). These could then be inactivated by further exposure to cre. **b.** Wild-type (WT) and 89-loxP MuHV-4 were tested for growth after low multiplicity inoculation of cre^+^ or cre^-^ NIH-3T3 cells (0.01 p.f.u./cell) and of cre^+^ or cre^-^ DCs (0.3 p.f.u./cell). Virus titers at each time point were determined by plaque assay of infected cell supernatants on BHK-21 cells. Equivalent data were obtained in 2 replicate experiments. **c.** Cre^+^ and cre^-^ NIH-3T3 cells were infected with wild-type (WT) or 8/9-loxP MuHV-4 (1 p.f.u./cell, 18 h). Infected cell DNA was digested with *Hin*DIII and hybridized with a probe flanking the 8/9 loxP site. Cre-mediated recombination was predicted to delete genomic co-ordinates 1–19055), thereby attaching the *Hin*DIII-restricted fragment detected by a 16237–19868 probe to the terminal repeats. Although residual unrecombined genomes hybridize better with the probe (3631 nt overlap), the 813 nt overlap with recombined genomes was sufficient to identify a terminal repeat ladder.

As a second approach to establishing the functional importance of DCs for host colonization, we generated a MuHV-4 mutant in which cre-mediated recombination would cause a lethal genomic deletion. Thus we inserted between ORFs 8 and 9 a loxP site compatible with that remaining at the genome left end after BAC cassette excision (Fig. 5a). Complete recombination between loxP sites would now excise both the BAC/eGFP cassette and the left end of the viral genome, including the essential ORFs 6, 7 and 8 [46]. It would also excise the M2 latency gene [47], the viral tRNA/miRNAs [48] and a likely promoter element for ORF73 [49], and so would probably also compromise the capacity of the viral genome to persist as a latent episome. Thus in contrast to eCFP excision from loxP-eCFP MuHV-4, which had no effect on viral fitness and so provided an index of virus exposure to cre recombinase, 8/9-loxP MuHV-4 would tell us the functional consequence of impaired DC infection.

To excise just the BAC cassette from 8/9-loxP MuHV-4 we passed it once through NIH-3T3-cre cells (2 p.f.u./cell) and selected viable eGFP^-^ clones. These grew normally in cre^-^ cells, but were severely attenuated for growth in cre^+^ fibroblasts (Fig. 5b). They were moderately attenuated for growth in cre^+^ DCs. To confirm the mechanism of attenuation we infected cre^-^ and cre^+^ fibroblasts with wild-type or 8/9-loxP MuHV-4, then isolated infected cell DNA, digested it with *Hin*DIII and probed it with a *Hin*DIII-N viral genomic clone that spans the ORF8/ORF9 junction [50]. Cre-mediated deletion of M1-ORF8 was predicted to remove the left end of the *Hin*DIII-N locus and join its remainder to the viral terminal repeats, thereby changing the probed fragment from 3632 bp to >20 kb (as MuHV-4 has up to 30 copies of its 1.2 kb terminal repeat unit), with a 1.2 kb submolar ladder due to variation in terminal repeat copy number. This is precisely what we observed (Fig. 5c).

### *In vivo* consequences of virus inactivation in DCs

**Figure 6:**
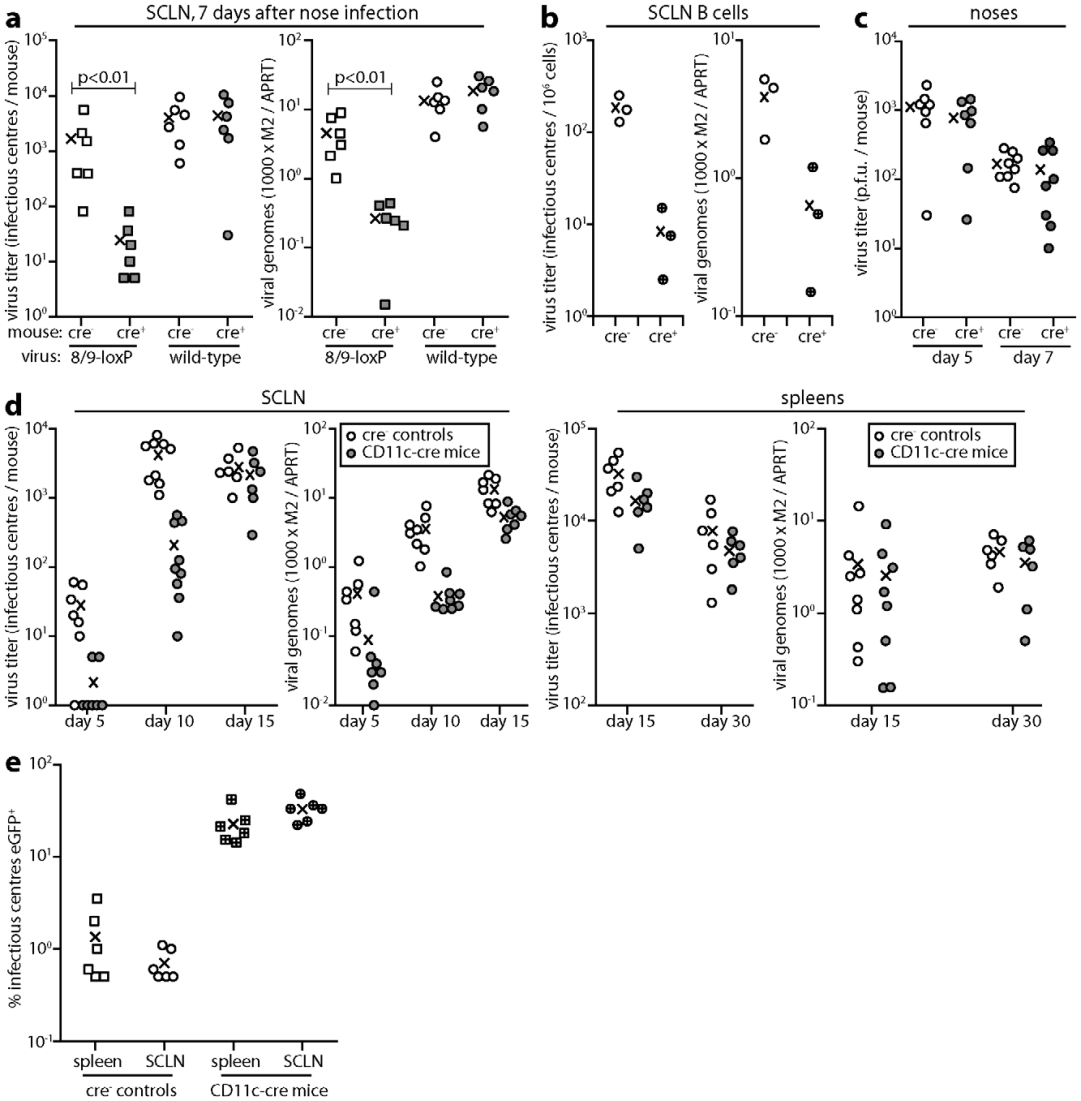
Colonization of CD11c-cre mice by 8/9-loxP MuHV-4. a. CD11c-cre mice (cre^+^) or non-transgenic littermate controls (cre^-^) were infected in the upper respiratory tract with 8/9-loxP or wild-type (WT) MuHV-4 (10^4^ p.f.u.). 7 days later SCLN were analyzed by infectious centre assay and Q-PCR. Viral genome loads (M2) were normalized by the host genome copy number (APRT) of each sample. Each point shows the result for 1 mouse. The crosses show means. 8/9-loxP MuHV-4 showed a significant defect in cre^+^ mouse infection (p<0.01 by Student's t test). Equivalent data were obtained in 3 replicate experiments. **b.** CD11c-cre mice (cre^+^) or non-transgenic littermate controls (cre^-^) were infected in the upper respiratory tract with 8/9-loxP MuHV-4 (10^4^ p.f.u.). 7 days later CD19^+^ B cells were recovered from the SCLN of pooled pairs of mice by flow cytometric sorting and analyzed by infectious centre assay and Q-PCR. Each point shows the result for 1 mouse. The crosses show means. 8/9-loxP MuHV-4 showed a significant, cre-dependent defect in both virus recovery from B cells (p<0.01 by Student's t test) and in B cell viral genome loads (p<0.05). **c.** Lytic titers of 8/9-loxP MuHV-4 were determined by plaque assay of noses after upper respiratory tract infection (10^4^ p.f.u.) of CD11c-cre mice or non-transgenic littermate controls. Each point shows the result for 1 mouse. The crosses show means. No significant difference in titer was observed between cre^+^ and cre^-^ mice. Equivalent data were obtained in 2 replicate experiments. **d.** CD11c-cre mice or non-transgenic controls were infected in the upper respiratory tract with 8/9-loxP MuHV-4 (10^4^ p.f.u.). SCLN and spleens were then analyzed by infectious centre assay and Q-PCR as in **a**. The colonization of cre^+^ mice was significantly reduced at days 5 and 10 post-infection (p<0.02 by Student's t test) but not at days 15 or 30. Equivalent data were obtained in 2 replicate experiments. **e.** CD11c-cre mice or non-transgenic controls were infected in the upper respiratory tract with a 1∶100 mixture of EF1a-eGFP and 8/9-loxP MuHV-4 (total 10^4^ p.f.u.). 30 days later latent virus was recovered from SCLN and spleens by infectious centre assay and typed as eGFP- or eGFP^+^. Each point shows the result for 1 mouse. Crosses show means. The proportion of recovered virus that was eGFP^+^ was significantly increased in cre^+^ versus cre^-^ mice (p<0.001 by Student's t test for each organ). Equivalent data were obtained in 2 replicate experiments.

8/9-loxP MuHV-4 showed a significant defect in SCLN colonization at day 7 after inoculation into the noses of CD11c-cre mice relative to non-transgenic controls, whereas wild-type MuHV-4 showed no difference (Fig. 6a). An independently derived 8/9-loxP mutant showed the same phenotype, while a revertant virus did not (Fig. S2). Therefore DC-specific virus attenuation reduced virus spread to lymph nodes. A similar cre-dependent defect was observed in the colonization of B cells, purified from SCLN by flow cytometric sorting (Fig. 6b). 8/9-loxP virus lytic titers, by contrast, were not significantly different between the noses of cre^+^ and cre^-^ mice (Fig. 6c), consistent with the limited loss of intergenic eCFP expression seen in noses (Fig. 4f).

Surprisingly, by day 15 post-infection (spleens and SCLN) the substantial early defect in SCLN colonization (Fig. 6d) of cre^+^ mice by 8/9-loxP MuHV-4 was no longer evident. MuHV-4 single gene knockouts [51], [52] have shown the same phenomenon of early replication defects not translating into differences in long-term latent loads. This possibly reflects that lower virus loads elicit less host response, such that mutant and wild-type viruses differ more in their speed of colonization than in their final set points. A different situation arises when mutant and wild-type viruses compete for a limited latency niche. To test this we co-infected cre^+^ and cre^-^ mice with a 1∶100 mix of EF1a-eGFP and 8/9-loxP viruses (Fig. 6e), and determined their relative levels of host colonization 30 days later by typing spleen and lymph node infectious centres as eGFP^-^ or eGFP^+^. The viruses recovered from non-transgenic mice showed the expected eGFP^+^:eGFP^-^ ratio of 1∶100. In contrast, those recovered from CD11c-cre mice showed ratios of 1∶3–1∶4, a 25-30-fold bias against 8/9-loxP MuHV-4. Therefore in the face of virus competition, an attenuation of DC infection substantially reduced host colonization.

### MuHV-4 alters the DC cytoskeleton

**Figure 7:**
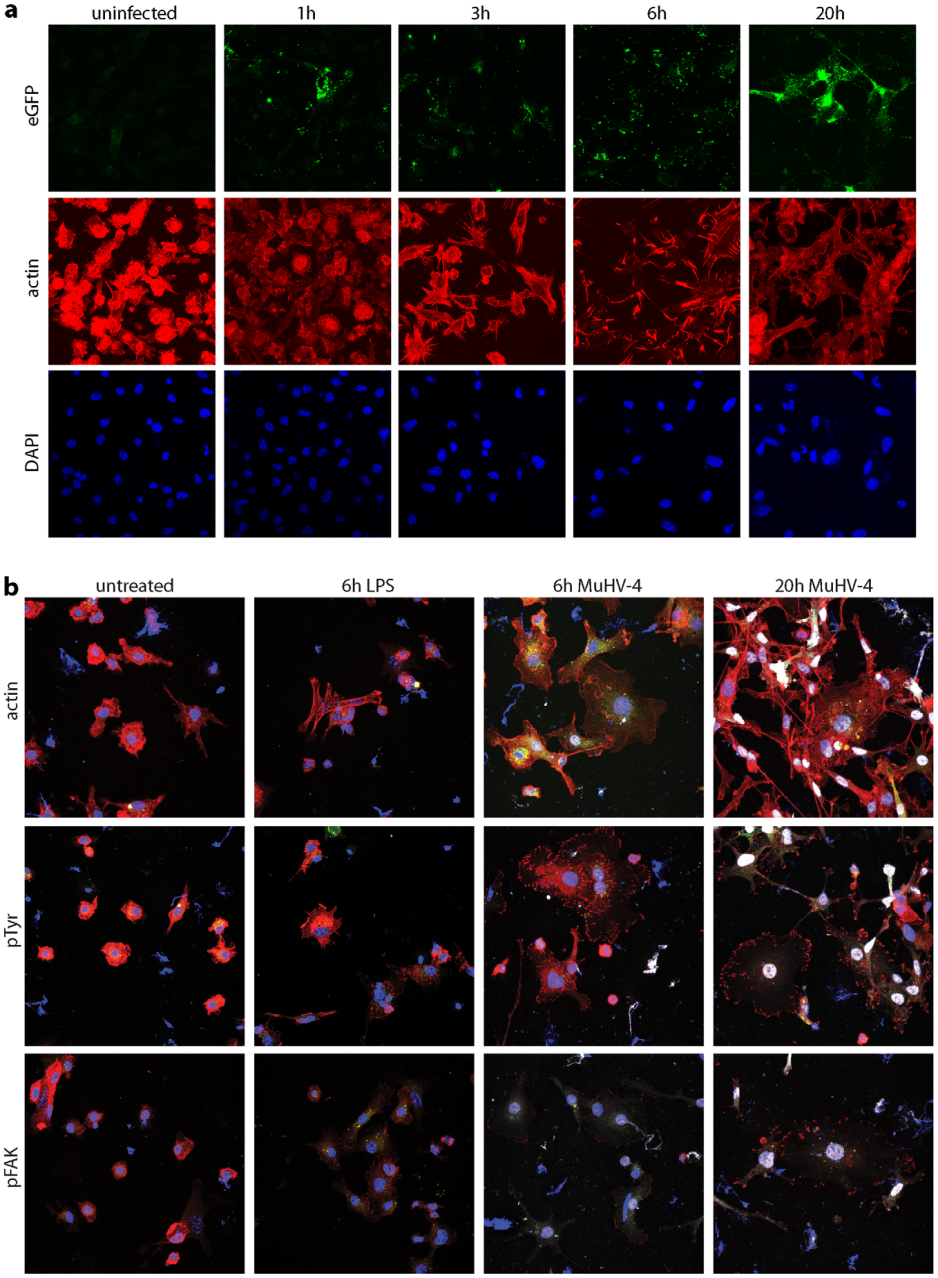
Modification of the DC cytoskeleton by MuHV-4. a. Bone marrow-derived DCs were left uninfected or exposed to gM-eGFP^+^ MuHV-4 (2 p.f.u./cell) for the time shown. The cells were then fixed, permeabilized and stained for actin with Alexa568-coupled phalloidin (red). EGFP fluorescence was visualized directly (green) and nuclei were counterstained with DAPI (blue). gM is a late lytic gene, so any eGFP signal seen before 20 h is likely to be from input virions. Equivalent data were obtained in 2 replicate experiments. **b.** Bone marrow-derived DCs were left untreated, exposed to 1 µg/ml LPS for 6 h, or exposed to EF1a-eGFP^+^ MuHV-4 (3 p.f.u./cell) for 6 h or 20 h. The cells were then fixed, permeabilized and stained for actin, phosphotyrosine (pTyr) or phosphorylated focal adhesion kinase (pFAK) (red). Nuclei were counter-stained with DAPI (blue) and eGFP fluorescence was visualized directly (white). Equivalent data were obtained in 2 replicate experiments.

DCs transport peripherally acquired antigens to lymph nodes even without viral infection [1]. Therefore MuHV-4 could reach lymph nodes simply by infecting DCs peripherally and remaining latent. However, viruses often alter cell behaviour to make host colonization more efficient. We looked for such effects using DCs grown from bone marrow stem cells with GM-CSF. After overnight MuHV-4 infection (3 p.f.u./cell), most bone marrow-derived DCs are latently infected - typically 20–30% express late lytic genes [45]. However almost all virus-exposed DCs showed cytoskeletal changes (Fig. 7a): actin was rapidly relocated to peripheral cytoplasmic projections, and after overnight infection the DCs had become flattened with many displaying long cytoplasmic processes. Phosphotyrosine and more specifically Y925-phosphorylated (activated) focal adhesion kinase (Fig. 7b) adopted peripheral, punctate distributions consistent with focal adhesion formation. MuHV-4-exposed DCs do not become non-specifically activated [44], [53], [54], and equivalent cytoskeletal changes were not induced by DC activation with lipopolysaccharide. Therefore these cytoskeletal changes were induced specifically by MuHV-4.

Real-time imaging (Video S1) showed that DCs became flattened and adherent within 6 h of exposure to MuHV-4, yet continued to show rapid changes in shape. This phenotype persisted at 20 h post-infection (Video S2), by which time the combination of adherence and dynamic remodelling and had led to the long cytoplasmic extensions seen in Fig. 7. Thus it appeared that infected DCs were motile but unable to detach from plastic. How these *in vitro* changes relate to *in vivo* DC migration is unclear, as tissue culture plastic provides a rather artificial surface, but it was clear that MuHV-4 actively manipulates the DC cytoskeleton.

## Discussion

Lymphotropic viruses arrive at mucosal epithelia, whereas naive lymphocytes circulate through organized lymphoid tissue. Therefore lymphotropic viruses face a problem in reaching their target cells. Lymphatic channels normally provide a route for DCs and cell-free antigens to travel from epithelia to lymph node subcapsular sinuses. Small, soluble antigens can pass directly into B cell follicles via specialized conduits [55]–[57], but larger antigens - immune complexes [58], virus-sized particles [59], and cell-free virions [60], [61] - are first captured by subcapsular sinus macrophages. Thus viruses can enter lymph nodes via migratory DCs or subcapsular sinus macrophages.

Most analysis of DC migration has depended on indirect measures such as T cell priming [62]. In contrast, virion capture by subcapsular sinus macrophages has been observed directly [60], [61]. However, direct imaging has depended on injecting large virion numbers. Such high doses may reveal mainly high-capacity rather than high-efficiency capture pathways, and the tissue pressures created by injection tend to force material into and along lymphatics. Therefore the relevance to non-invasive infections of antigen injections must still be established. Here we analyzed lymph node colonization after a non-invasive infection. MuHV-4 does not establish a detectable cell-free viraemia [63], and depends for its *in vivo* propagation on cell/cell spread [64] more than on cell-free virion binding [65] or release [66]. Thus it might be expected to follow a cell-associated route to lymph nodes. Consistent with this idea, we identified a major role for DCs in passing infection to B cells. Thus MuHV-4 sets a new precedent for host exploitation by a lymphotropic virus.

We could not determine whether MuHV-4 reaches lymph nodes only via DCs, but the similar efficiencies of eCFP excision and 8/9-loxP virus attenuation between *in vitro* DC infection and *in vivo* host colonization argued that DC infection plays a predominant role. This would also be consistent with MuHV-4 still infecting the lymph nodes of B cell-deficient mice [67]. CD11c is a well-established DC marker, but is not exclusive to DCs [68]. Expression on activated T cells can be discounted here as MuHV-4 does not infect T cells. Lung macrophages express CD11c [68], as do subcapsular sinus macrophages at a low level [60]. However lysM-cre mice, which express cre in macrophages [69], showed substantial eCFP loss from loxP-eCFP MuHV-4 only after lower respiratory tract infection (unpublished data). Therefore while macrophages may feature prominently in MuHV-4 lung infection, DCs provided the major route of its transfer from nose to lymph nodes. Thus MuHV-4 exploits olfaction to enter the upper respiratory tract [29]; lymphocytes to persist [70]; and DCs to link them by virus transport.

Cells migrate by forming cytoplasmic protrusions, adhering these to the extracellular matrix, then detaching and contracting their trailing edges [71]. Actin, focal adhesion kinase and tyrosine phosphorylation all play central roles, so the redistributions of these markers in infected DCs, independent of viral lytic gene expression, was consistent with MuHV-4 inducing latently infected DCs to migrate. ORF27/ORF58-dependent actin rearrangements [72] could then promote further virion spread upon lytic reactivation. However, *in vitro* DC migration was prevented by infected DCs not detaching from plastic, and we cannot exclude that the infected SCLN DCs were resident and infected through antigen capture, rather than being migratory. Therefore virus-induced DC migration needs further investigation; current evidence establishes only that DC infection is important for establishing B cell infection in lymph nodes.

MuHV-4-infected DCs may also promote the amplification of B cell infection by secreting the M3 chemokine binding protein to protect *in trans* against CD8^+^ T cell attack [73]. In both this setting and that of virus transfer, the B cell latency defects of MuHV-4 lacking its MHC class I down-regulation gene K3 [51] or its bcl-2 homolog M11 [52] could reflect that these genes function in DCs [74], [75]. Exploiting DCs presumably brings gamma-herpesviruses advantages of efficiency and stealth. Whether it also creates possibilities for therapeutic intervention remains to be seen.

## Materials and Methods

### Mice

All animal experiments were approved by the Cambridge University ethical review board and by the UK Home Office (PPL 80/1992), and carried out in accordance with the Animals (Scientific Procedures) Act 1986. C57BL/6 and BALB/c mice were obtained from Harlan U.K. C57BL/6 back-crossed CD11c-cre mice, which express cre recombinase in DCs [76], were obtained from Jackson Laboratories and maintained as heterozygote × C57BL/6 non-transgenic crosses. Mice were typed by PCR of tail DNA, using the primers 5′-ACTTGGCAGCTGTCTCCAAG, 5′-GCGAACATCTTCAGGTTCTG (transgene-specific) and 5′ CAAATGTTGCTTGTCTGGTG, 5′- GTCAGTCGAGTGCACAGTTT (internal control). Mice were infected with MuHV-4 when 6–12 weeks old. Intranasal infections with anaesthesia were in 30 µl; those without were in 5 µl. For luciferase imaging, mice were injected intraperitoneally with luciferin (2 mg/mouse), anaesthetized with isoflurane, then scanned with an IVIS Lumina (Caliper Life Sciences). To image specific tissues mice were killed and dissected after luciferin injection. All experiments conformed to local animal ethics regulations and Home Office Project Licence 80/1992.

### Cells

Baby hamster kidney (BHK-21) cells, NIH-3T3 cells and NIH-3T3-CRE cells [50] were propagated in Dulbecco's modified Eagle medium (Invitrogen Corporation) supplemented with 2 mM glutamine, 100 U/ml penicillin, 100 mg/ml streptomycin and 10% fetal calf serum (complete medium). Dendritic cells were derived from bone marrow progenitors of CD11c-cre mice or non-transgenic litter-mates. Bone marrow cells were depleted of adherent cells (30 min, 37°C) and then cultured in RPMI with 10% fetal calf serum, 50 µM 2-mercaptoethanol, 100 U/ml penicillin, 100 mg/ml streptomycin and 7.5 ng/ml GM-CSF (PeproTech). The medium was changed every 2 d, and non-adherent cells harvested after 7 d. These were >90% GR-1^-^CD11c^+^ by flow cytometry.

### Bead-based cell separation

Lymph nodes were removed post-mortem, finely minced, digested (20 min) with type II collagenase (1 mg/ml, Worthington Biochemicals) plus DNaseI (20 µg/ml, Boehringer Mannheim), then incubated (5 min) in 100 mM EDTA to disrupt cell/cell conjugates. Debris was removed by filtration (100 µm). B cells, DCs and macrophages were then separated with antibody-coated magnetic beads (Miltenyi Biotec). First B cells were selected with anti-CD45R, then DCs were selected with anti-CD11c, then macrophages were selected with anti-CD11b. Each population was >90% pure by flow cytometry.

### Viruses

Luciferase^+^ [29], EF1a-eGFP^+^ (May and Stevenson, 2010), and gM-eGFP^+^ [77] MuHV-4 reporter viruses have been described. To insert a floxed eCFP expression cassette into the MuHV-4 genome, the eCFP coding sequence was amplified by PCR (*Pfu* ploymerase, Promega Corporation) from pMSCV-IRES-eCFP using *Eco*RI and *Kpn*I-restricted primers which also incorporated modified loxP sites. Specifically the GCATACAT spacer region was changed to GTATACAT [78] = loxP*. The loxP*-flanked eCFP coding sequence was then cloned as an *Eco*RI/*Kpn*I-restricted fragment into the corresponding sites of pSP73-M3-pA [79]. This placed it between a 500 bp MuHV-4 M3 promoter and a bovine growth hormone polyadenylation site. The M3-driven eCFP expression cassette was then excised from pSP73-M3-pA with *Bgl*II+*Xho*I, blunted with Klenow fragment DNA polymerase and cloned into the blunted *Mfe*I site (genomic co-ordinate 77176) [80] of a *Bgl*II MuHV-4 genomic clone (co-ordinates 75338–78717), again in pSP73. This placed it between the 3′ ends of ORFs 57 and 58. The eCFP expression cassette plus genomic flanks was then subcloned as a *Bgl*II fragment into the *Bam*HI site of the pST76K-SR shuttle vector and recombined into a MuHV-4 BAC [44].

To insert a loxP site into the MuHV-4 genome between ORF8 (genomic coordinates 16526–19054) and ORF9 (genomic coordinates 19217–22300), we amplified by PCR 2 genomic flanks around genomic coordinate 19055, generating the loxP site from overlapping 3′ extensions of the inner primers. The 2 PCR products were then mixed and re-amplified with the outer primers to generate a single product. A *Sma*I/*Bgl*II-restricted fragment, corresponding to genomic coordinates 18614–19424 with the new loxP site at 19055, was then excised from this product, cloned into the *Sma*I/*Bam*HI sites of pST76K-SR, and recombined into the MuHV-4 BAC. Mutant viruses were identified by restriction enzyme mapping and by DNA sequencing across the insertion site. We also derived a revertant virus by shuttle vector-mediated reconstitution of the original genome region without a loxP site. BAC DNA was reconstituted into infectious virus by transfection into BHK-21 cells (Fugene-6, Roche Diagnostics). The floxed BAC/GFP cassette was removed by virus passage through NIH 3T3-CRE cells, followed by plaque purification. Virus stocks were prepared in BHK-21 cells. Infected cell debris was removed by centrifugation (400×*g*, 3 min), and virions then recovered from supernatants by ultracentrifugation (38000×*g*, 90 min).

### Infectivity assays

Virus stocks were titered by plaque assay on BHK-21 cells [66]. Cell monolayers were incubated with virus dilutions (2 h, 37°C), overlaid with 0.3% carboxymethylcellulose, and 4 days later fixed in 4% formaldehyde and stained with 0.1% toluidine blue for plaque counting. Infectious virus in lungs and noses was measured by freeze-thawing the tissues, then homogenizing them in 1 ml complete medium prior to plaque assay. Latent virus was measured by infectious centre assay [66]: spleen cells were co-cultured with BHK-21 cells, then fixed and stained for plaque counting after 4 days. Plaque assay titers of freeze-thawed lymphoid homogenates were always <1% of infectious center assay titers, so the latter essentially measured reactivable latent virus. To distinguish eCFP^+^ and eCFP^-^ (or eGFP^+^ and eGFP^-^) viruses, plaque or infectious centre assays were performed in limiting dilution format. Each well was then scanned under normal illumination, and for each positive well a chosen plaque scored as fluorescent or not under ultra-violet illumination. Thus each positive well was counted just once.

### Viral genome quantitation

MuHV-4 genomic co-ordinates 4166–4252, corresponding to the M2 locus, was amplified from tissue DNA (50–80 ng) by quantitative PCR (Rotor Gene 3000, Corbett Research). The PCR products were quantitated by hybridization with a Taqman probe (genomic coordinates 4218–4189) and converted to genome copies by comparison with a standard curve of cloned plasmid template amplified in parallel [81]. Cellular DNA was quantitated in parallel by amplifying part of the adenosine phosphoribosyl transferase gene.

### Southern blotting

Viral DNA was extracted by alkaline lysis [66], digested with *Hin*DIII, electrophoresed and transferred to nylon membranes (Roche Diagnostics). A ^32^P-dCTP labelled probe (APBiotech) was generated by random primer extension (DECA prime II kit, Ambion). Membranes were hybridised with probe (65°C, 18 h), washed in 30 mM sodium chloride/3 mM sodium citrate/0.1% sodium dodecyl sulfate at 65°C and exposed to X-ray film.

### Flow cytometry

Cells infected with eCFP^+^ or GFP^+^ viruses were trypsinized, washed in PBS and analysed directly for eGFP and eCFP fluorescence on a Fortessa flow cytometer (BD Biosciences). For specific staining of lymph node cells, IgG Fc receptors were blocked by pre-incubation (30 min, 4°C) with unlabelled rat anti-CD16/32 mAb, then stained for CD19, CD69, MHC class II or syndecan-4 with phycoerythrin-conjugated rat mAbs (all from BD Biosciences) and for surface immunoglobulin with Alexafluor 633-conjugated goat anti-mouse Ig(H+L) pAb (Invitrogen). After washing, cells were analysed on a FACS Calibur using Cellquest (BD Biosciences) and Weasel (Walter and Eliza Hall Institute of Medical Research). For flow cytometric sorting, spleen cells were pooled from pairs of mice and stained with phycoerythrin-conjugated rat anti-mouse CD19 mAb and Alexafluor 633-conjugated goat anti-mouse Ig(H+L) pAb. CD19^+^Ig^+^ cells were selected with a FACS Vantage (BD Biosciences). The recovered cells were >98% pure.

### Immunofluorescence

DCs were plated overnight onto poly-D-lysine-coated coverslips after 7 days of culture, then infected or not with MuHV-4. After further culture, the cells were washed with PBS, fixed in 2% formaldehyde, permeabilized with 0.1% Triton-X-100, blocked with 3% BSA and stained for actin with Alexafluor568-conjugated phalloidin (Invitrogen), for phospho-tyrosine with mAb PY99 (Santa Cruz Biotechnology) plus Alexafluor568-conjugated goat anti-mouse pAb (Invitrogen), and for Y925-phosphorylated focal adhesion kinase with ab38512 (AbCam) plus Alexafluor568-conjugated goat anti-rabbit pAb (Invitrogen). EGFP fluorescence was visualized directly. After washing in PBS, the cells were mounted in Prolong Gold with DAPI (Invitrogen) and imaged with a Leica Confocal microscope.

Lymph nodes were removed post-mortem, fixed (24 h, 4°C) in 1% formaldehyde/10 mM sodium periodate/75 mM L-lysine, then equilibrated in 30% sucrose, and frozen in OCT mounting medium. 5 µm sections were blocked with 2% goat serum then stained for CD45R (mAb RA3-6B2, B cells) or for CD11c (mAb HL3, DCs) (BD Biosciences) plus Alexafluor568 or 488-conjugated goat anti-rat or anti-hamster IgG pAb (Invitrogen). Infected cells were detected with rabbit anti-eGFP pAb (Abcam) plus Alexafluor488 or 568-conjugated goat anti-rabbit IgG pAb (Invitrogen). The sections were mounted in Prolong Gold (Invitrogen) and imaged with a Leica Confocal microscope.

## Supporting Information

Figure S1 **Replication of loxP-eCFP MuHV-4 in cre^-^ mice. a.** C57BL/6 mice were infected in the upper respiratory tract with wild-type or loxP-eCFP MuHV-4 (10**^4^** p.f.u.). 6 days later noses were titered for infectious virus by plaque assay. Each point shows the result for 1 mouse. The crosses show means. **b.** C57BL/6 mice were infected as in a. 16 days later spleens were titered for recoverable latent virus by infectious centre assay. Each point shows the result for 1 mouse. The crosses show means. LoxP-eGFP MuHV-3 showed no significant defect in either lytic or latent infection.
(TIF)

Figure S2 **SCLN infection of cre^+^ mice.** CD11c-cre mice were infected in the upper respiratory tract with wild-type (WT) MuHV-4, the 8/9-loxP mutant, an independently derived mutant (ind) or a revertant virus (rev) (all 10**^4^** p.f.u.). 7 days later SCLN were analyzed for virus colonization by infectious centre assay. Each point shows the result for 1 mouse. The crosses show means. The 8/9-loxP mutants both showed a significant infection defect (p<0.002 by Student's t test), whereas the revertant virus did not (p = 0.48).
(TIF)

Video S1 **Bone marrow-derived DCs were infected with gM-eGFP^+^ MuHV-4 (3 p.f.u./cell) and imaged 6 h later by time-lapse confocal microscopy (Leica).** Images were recorded every 8 sec over 7 min. The left-hand panel shows eGFP fluoresence and the right-hand panel the corresponding phase contrast image. At 6 h after exposure to MuHV-4, DCs show little late lytic gene expression. Thus the eGFP signal comes from the input virions. The non-adherent DCs correspond to those more activated prior to virus exposure, which are poorly infected and so remain eGFP^-^. The DCs taking up virions, and presumably becoming infected, show flattening, adherence to plastic and marked cytoplasmic motility.
(AVI)

Video S2 **Bone marrow-derived DCs were infected as in Video S1 and imaged 20 h later.** Images were recorded every 8 sec over 7 min. The left-hand panel shows eGFP fluoresence and the right-hand panel the corresponding phase contrast image. The infected DCs (eGFP^+^) remain dynamic, consistent with actin remodelling, but are firmly adherent to plastic. This has led to the formation of long cytoplasmic processes.
(AVI)
