# Supplementary figures and images for: Murid Herpesvirus-4 Exploits Dendritic Cells to Infect B Cells
Source: PLoS Pathog. 2011 Nov 10;7(11):e1002346. doi: 10.1371/journal.ppat.1002346 (PMC3213091; doi:10.1371/journal.ppat.1002346)

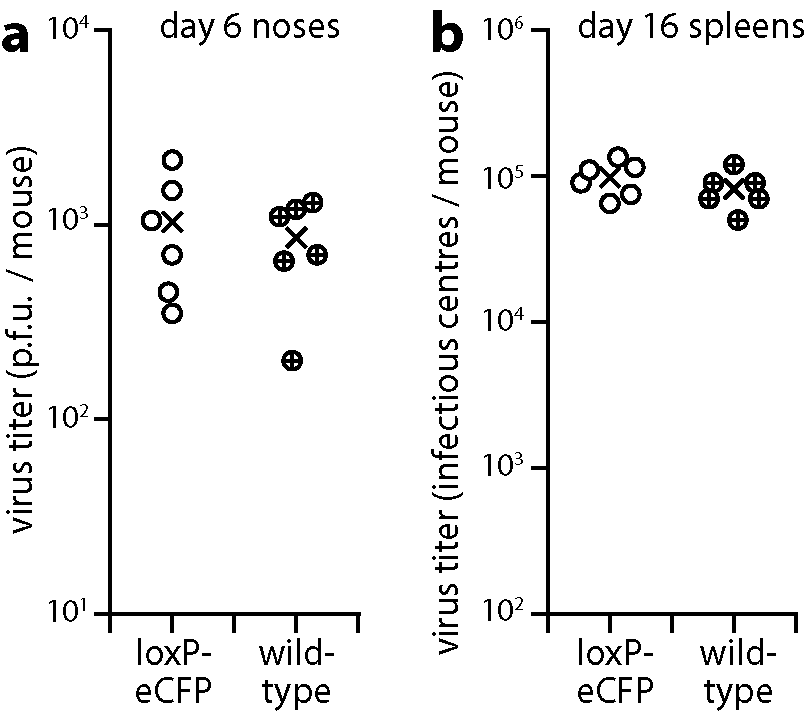

Supplement: Figure S1 — Replication of loxP-eCFP MuHV-4 in cre- mice. a. C57BL/6 mice were infected in the upper respiratory tract with wild-type or loxP-eCFP MuHV-4 (104 p.f.u.). 6 days later noses were titered for infectious virus by plaque assay. Each point shows the result for 1 mouse. The crosses show means. b. C57BL/6 mice were infected as in a. 16 days later spleens were titered for recoverable latent virus by infectious centre assay. Each point shows the result for 1 mouse. The crosses show means. LoxP-eGFP MuHV-3 showed no significant defect in either lytic or latent infection. (TIF) [file ppat.1002346.s001.tif]

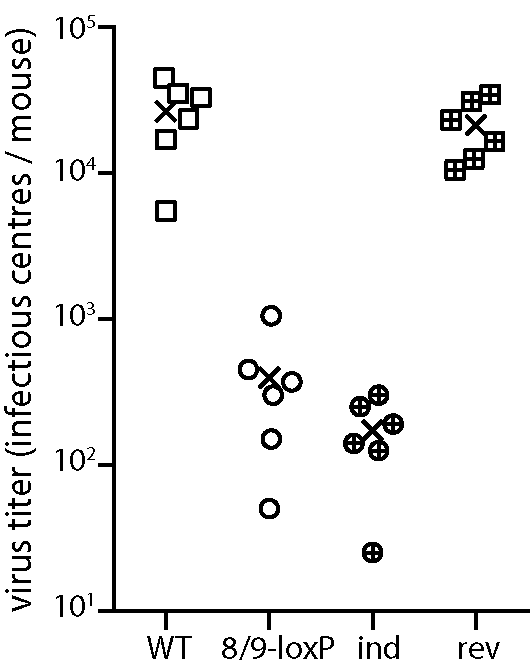

Supplement: Figure S2 — SCLN infection of cre+ mice. CD11c-cre mice were infected in the upper respiratory tract with wild-type (WT) MuHV-4, the 8/9-loxP mutant, an independently derived mutant (ind) or a revertant virus (rev) (all 104 p.f.u.). 7 days later SCLN were analyzed for virus colonization by infectious centre assay. Each point shows the result for 1 mouse. The crosses show means. The 8/9-loxP mutants both showed a significant infection defect (p<0.002 by Student's t test), whereas the revertant virus did not (p = 0.48). (TIF) [file ppat.1002346.s002.tif]
